# Fatty Acid Composition of *M. Biceps Femoris* of Edible Dormouse (*Glis glis* L.)

**DOI:** 10.3390/ani12233284

**Published:** 2022-11-25

**Authors:** Lana Pađen, Susana P. Alves, Rui J. B. Bessa, André M. Almeida, Miljenko Bujanić, Dean Konjević

**Affiliations:** 1 Department of Physiology and Radiobiology, Faculty of Veterinary Medicine, University of Zagreb, 10000 Zagreb, Croatia; 2 CIISA/FMV–Centro de Investigação Interdisciplinar em Sanidade Animal, Faculdade de Medicina Veterinária, Universidade de Lisboa, 1300-477 Lisboa, Portugal; 3 Laboratório Associado Para Ciência Animal e Veterinária (AL4AnimalS), 1300-477 Lisboa, Portugal; 4 LEAF—Linking Landscape, Environment, Agriculture and Food Research Center, Associated Laboratory TERRA, Instituto Superior de Agronomia, Universidade de Lisboa, Tapada da Ajuda, 1349-017 Lisboa, Portugal; 5 Department of Veterinary Economics and Epidemiology, Faculty of Veterinary Medicine, University of Zagreb, 10000 Zagreb, Croatia

**Keywords:** fatty acid composition, *Glis glis*, feeding habits, *m. biceps femoris*

## Abstract

**Simple Summary:**

The consumption of edible dormouse (*Glis glis* L.) meat is accepted as part of the tradition and heritage of certain regions of southern Europe. Since the literature regarding the composition of dormouse meat is scarce and no studies reported the fatty acid composition of muscle, this study aimed to investigate the fatty acid composition of edible dormouse *m. biceps femoris* in both sexes. Furthermore, some fatty acids suggest feeding of an edible dormouse on a specific group of plants. This work can contribute to increase the knowledge on edible dormouse physiology and nutritional traits as well as to update food composition database.

**Abstract:**

This study aimed to investigate the fatty acid (FA) composition of edible dormouse *m. biceps femoris* in both sexes. More than 20 FA were identified in the muscle, with the 18:1*cis*-9 (oleic acid) being the most abundant in both sexes, comprising more than 50% of total FA in muscle. The most dominated FA were monounsaturated (MUFA), followed by saturated FA (SFA) and polyunsaturated FA (PUFA), reaching 54.8%, 25.43% and 19.8% of total FA, respectively. Sums of PUFA and n-3 PUFA tended (*p* > 0.05) to be higher in males than in females. There were no significant differences between sexes on the FA composition. Nevertheless, the 18:2n-6 tended to differ between sexes (*p* = 0.063). Several long-chain PUFA (LC-PUFA) were detected in dormouse muscle, with the 20:4 n-6 (arachidonic acid, AA) and the 22:6 n-3 (docosahexaenoic acid, DHA) being the most abundant in both sexes. The relatively high stearoyl-CoA desaturase (SCD) indexes and the large concentration of 18:1*cis*-9 in dormouse muscle tissues might point to a low mobilization of the SCD products. Furthermore, finding the unusual FA 20:3 ∆5,∆11,∆14, suggests feeding on leaf and wood lipids of Coniferophytes. We demonstrated sexual size monomorphism in edible dormouse. The literature regarding the composition of dormouse meat is scarce and no studies reported the FA composition of muscle, thus, this work can contribute to increasing the knowledge on edible dormouse physiology and nutritional traits.

## 1. Introduction

The Edible or Fat Dormouse (*Glis glis* L.) is a member of the order *Rodentia*, family *Gliridae*, and the genus *Glis* [1]. The edible dormouse is a medium-sized mammal that can weight up to 400 g and grow to a length of about 30 cm, including the up-to-15 cm tail [2]. It can be found across Europe in both temperate and Mediterranean forests from Spain to the Volga, including major Mediterranean islands such as Crete, Sicily, and Sardinia. According to literature [3], some regions of Europe recorded declines in numbers, probably due to unorganized logging and poor forest management. Such a situation has resulted in the protection of the edible dormouse in many European countries, whereas in some, it is included in the Red book of threatened species [4]. However, the real cause of the population decline is still unknown and is probably more complex [5].

**Figure 1:**
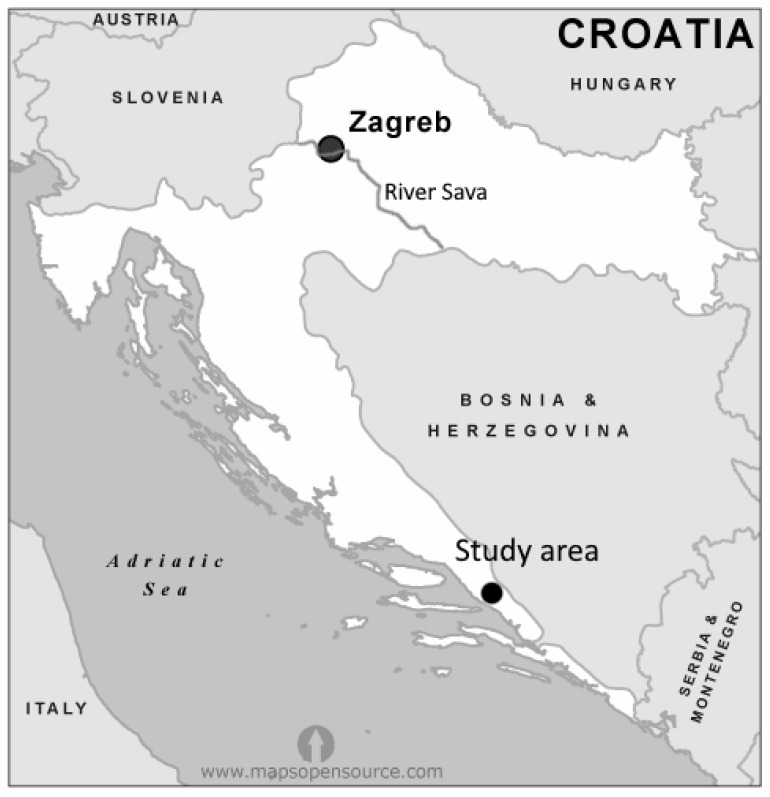
Study area in Croatia, Dalmatian hinterland region.

The edible dormouse is distributed over the whole territory of Croatia [6] and is protected by the Hunting Law [7], which allows a hunting period from 16th September until the 1st December, using the special dormice traps and natural bait. Hunting is permitted only south of the Sava River, in contrast to the north part, where the edible dormouse is protected throughout the entire year (Figure 1). Dalmatia is a narrow strip of land on the eastern shore of the Adriatic Sea. The most frequent dormouse habitats are forest associations of *Querco pubescenti-Carpinetum orientalis* [8] and *Fraxino orni-Quercetum ilicis* [9], in areas with distinct karst micro-relief or with rock walls that dominate the landscape. At higher altitudes, beech (*Fagus sylvatica*) and the common juniper (*Juniperus communis*) can be found [10]. The management of edible dormice in Croatia is done in a sustainable way [11]. Indeed, a permit is needed for dormouse hunting, which is issued by the hunting concession holder of a hunting ground, or by the hunting ground owner, based on an approved game management plan [4].

In central and northern Europe, beechnut and acorn are edible dormice major food sources, although sometimes temporarily limited in availability [12,13,14]. The edible dormouse is a hibernating animal; an adult individual can show quite pronounced seasonal body mass variations, as well as accumulation of large quantities of adipose tissue prior to hibernation [12,15]. Older animals can weight up to 400 g before hibernation [5]. In contrast, younger animals are lighter and will stay awake longer to accumulate more fat for winter dormancy [16]. The edible dormouse hibernates in natural cavities in beech, fir, and mountain maple old trunks [17]. After hibernation, the edible dormouse is first observed in nature from mid-April or May, depending on the climate and altitude. A variety of fruits, forest seeds, young birds, and eggs are often chosen as food, and various species of grass and pine needles predominate in order to cleanse their digestive systems before hibernation [18]. However, when available, beech nuts represent the main food source [19].

Dormice hunting in Gorski kotar region in Croatia is a long tradition dating from Middle Ages, when the first settlers came into the area at the time of the Dukes of Krk, the Frankopans. Dormouse meat, which was salted and skinned, was put into wooden containers and kept in cold rooms to ensure that there was “food of the Gods” (*deorum cibus*) in long snowy winters [4]. Dormouse mat was seen as a special delicacy by Romans [11], and furthermore, the animal’s perirenal and subcutaneous adipose tissue was used as a traditional ointment for the healing of wounds, burns, and skin diseases. Dormouse meat consumption is accepted as part of the tradition and heritage of certain regions of Croatia, where it is believed that adipose tissue has healing properties when included in the diet [20]. Despite that, the literature is scarce regarding the composition of dormouse meat, and to the best of our knowledge, no studies have reported the fatty acid (FA) composition of muscle and especially the nutritional value of dormice meat with regard to human health. Apart from the potential significance for human health, dormice can harbor some pathogens, like *Borrelia burgdorferi* in Croatia [21].

In line with what was stated above, this study aimed to investigate the FA composition of the edible dormouse hind leg muscle, thus contributing to increasing the knowledge in edible dormouse physiology and nutritional traits.

## 2. Materials and Methods

The study was conducted on 30 individuals of edible dormouse (*Glis glis*; 16 females, 14 males) collected during the regular hunting season in the period from 16 September to 30 November 2017. The age of the captured animals was not explicitly determined, but all animals were categorized as young adults because of their low live weight (around 120 g) at the autumn season. Prior to sampling, animals were weighted with the tail and without the tail. Body mass of edible dormouse was 113.4 ± 22.9 g for females and 126.2 ± 30.2 g for males.

This study was conducted in the Dalmatian hinterland region, as shown in Figure 1. In terms of relief, the research area belongs to the lowland type. The peculiarity of the relief is that the central part is dominated by the valley, whereas on the northwest, north, northeast, and southeast sides the terrain is hilly, with altitude heights from 200 m at the Great Prologue to 480 m below Gradina hills. These are typical landscapes features of the eastern Adriatic karst hinterland. The sloping terrains are dominated by skeletal soils, and in the valleys, there is a layering of the earth in a thicker layer [7]. The dormice traps were placed in the tree canopy at a height of about 3 m, secured with the hazel rod. An apple or carob is usually used as bait in the trap. Recent efforts have been focused on making traps that would selectively save lesser, and consequently, younger specimens [4,5]. Upon collection, animals were frozen on −20 °C until sampling. Sampling was conducted during October and November 2017 at the Department of Veterinary Pathology Faculty of Veterinary Medicine University of Zagreb. Whole *m. biceps femoris* was dissected with a scalpel and stored at −20 °C. The samples were then freeze-dried for 72 h until constant weight using a SCANVAC CoolSafe 55-4, freeze drier (Labogene ApS, Lynge, Denmark) at the Animal Production Systems Lab of the Interdisciplinary Centre of Research in Animal Health, Faculty of Veterinary Medicine-University of Lisboa (Portugal).

Lipids from lyophilized muscle were extracted using the method of [22] with dichloromethane and methanol (2:1, *v*/*v*) instead of chloroform and methanol. Total lipids were measured gravimetrically, in duplicate, by weighting the fatty residue obtained after solvent evaporation. Fatty acid methyl esters (FAME) were prepared from the lipid extracts by a basic followed by acid transesterification procedure, as described by [23]. Briefly, 1 mL of toluene was added to the lipid extract, then 3 mL of sodium methoxide in methanol (0.5 M) was added and left to react for about 30 min at 50 °C; another 2 mL of HCl in methanol (1.25 M) was added to the reaction vessel, which was left to react for a further 10 min at 80 °C. After cooling, 2 mL of 6% aqueous potassium carbonate was added to the reaction tube and FAME were extracted with 4 mL of hexane. The solvent was removed under a flow of nitrogen at 37 °C, and the final residue was dissolved in 1 mL of hexane and stored at −20 °C until gas chromatography (GC) analysis.

FAME were quantified by GC with flame ionization detection (GC-FID) using a Shimadzu GC-2010 Plus chromatograph (Shimadzu, Kyoto, Japan) equipped with a SP-2560 capillary column (100 m, 0.25 mm i.d., 0.20 μm film thickness, Supelco, Bellefonte, PA, USA). Helium was used as carrier gas at a constant flow of 1 mL/min, and the injector and detector temperatures were 220 and 250 °C, respectively. Column oven programmed temperatures were as follows: initial oven temperature of 50 °C was held for 1 min, increased to 150 °C at 50 °C/min and held for 20 min, then increased to 190 °C at 1 °C/min, and finally increased to 220 °C at 2 °C/min and maintained for 30 min. Identification of FAME was achieved by comparing the FAME retention times with those of commercial standards (FAME mix 37 components from Supelco Inc., Bellefonte, PA, USA. The notation “n-number” was used for the all polyunsaturated FA, with all cis methylene interrupted double, where the n-number indicates the position of the first double bond counted from the methyl terminal end of the carbon chain.

Stearoyl-CoA desaturase (SCD) activity indices were estimated by computing the ratio of product/(substrate + product) SCDi-14 = [14:1c9/(14:1c9 + 14:0)] × 100; SCDi-16 = [16:1c9/(16:1c9 + 16:0)] × 100; SCDi-17 = [17:1c9/(17:1c9 + 17:0)] × 100); SCDi-18 = [18:1c9/(18:1c9 + 18:0)] × 100.

The FA composition in muscle was analyzed using the software STATISTICA (data analysis software system), version 12.0 (StatSoft, Tulsa, OK, USA) with a model that included the sex (female vs. male) as the single effect. In addition, differences on the FA sums among tissues were analyzed, considering the tissue as a single effect. Significance was declared at *p* ≤ 0.05 and tendency at *p* < 0.1, and data is presented as least square means and standard error of the mean (SEM). The correlation between study parameters (Spearman Rank Order Correlation was used when distribution of parameters was not normal, whereas Pearson correlation coefficient was used in cases of normal distribution) was tested using the same statistical software. The level of statistical significance was set at *p* ≤ 0.05.

## 3. Results

### 3.1. Body Mass and Fatty Acid Composition of Muscle

Male weights including tail were on average 11.3% heavier than females with tail; on the other hand, males without the tail were only 11% heavier than females without the tail. There was no significant difference (*p* > 0.05) between sexes. *M. biceps femoris* of edible dormouse contained about 163 mg/g DM of intramuscular fat, with no differences between sexes (*p* = 0.506). Regarding the FA composition, more than 20 FA were identified in the muscle (Table 1), with the 18:1*cis*-9 (oleic acid) being the most abundant in both sexes, comprising more than 50% of total FA in muscle. The 18:1*cis*-9 together with the 16:0 (palmitic acid) and 18:0 (stearic acid) comprise more than 74% of total FA in muscle. There were no significant differences between sexes on the FA composition (Table 1). Nevertheless, the 18:2n-6 tended to differ between sexes (*p* = 0.063). Several long-chain PUFA (LC-PUFA) were detected in dormouse muscle, with the 20:4 n-6 (arachidonic acid, AA) and the 22:6 n-3 (docosahexaenoic acid, DHA) being the most abundant in both sexes. An unusual ∆5-olefinic FA, i.e., the 20:3 ∆5,∆11,∆14, was detected in dormouse muscle comprising 0.1% of total FA.

### 3.2. Nutritional Fatty Acid Sums and Ratios

The FA sums and ratios with nutritional interest in dormouse muscle are presented in Table 2. The FA in the muscle of dormice was dominated by MUFA (54.8% of total FA), followed by SFA (25.43% of total FA) and PUFA (19.8% of total FA). There were no relevant differences in the FA sums between females and males. However, the sum of PUFA and n-3 PUFA tended to be higher in males than in females (*p* = 0.099, *p* = 0.058, respectively). Regarding the ratios, no significant differences were observed between males and females, however the PUFA/SFA tended (*p* = 0.059) to be higher in males than in females, whereas the 18:1/18:2 and AA/DHA ratios tended to be higher in females compared to males.

### 3.3. Stearoyl-CoA Desaturase Activity Indices

**Figure 2:**
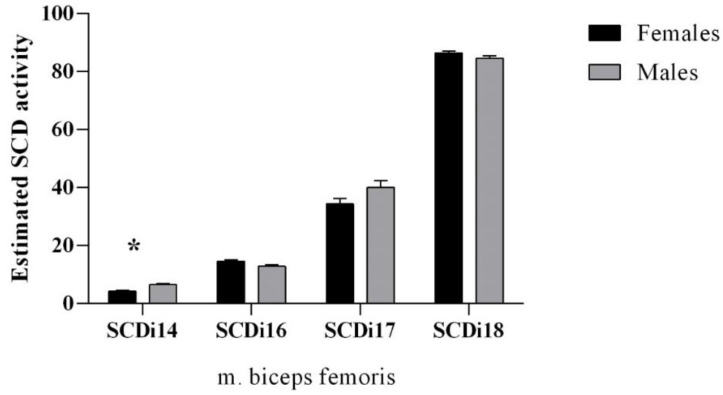
Stearoyl-CoA desaturase activity indices of edible dormouse (*Glis glis*) muscle (*m. biceps femoris*). * statistical significance at *p* < 0.05.

The SCD enzyme activity indices are presented in Figure 2. The SCDi-14 was greater in males compared to females (*p* = 0.027). No differences between sexes (*p* > 0.05) were observed in the other estimated SCD activity indices.

### 3.4. Figures and Tables

In this study, we found an inversely proportional relationship between ARA (females, r = −0.93, *p* < 0.05; males, r = −0.94, *p* < 0.05), DHA (females, r = −0.95, *p* < 0.05, males, r = −0.95, *p* < 0.05), and muscle lipid contents, whereas 18:1*cis*-9 and lipid contents showed a proportional relationship (females, r = 0.88, *p* < 0.05; males, r = 0.84, *p* < 0.05). We also found an inversely related ratio of 20:4n-6/20:3n-6 and body mass in males as well as an inversely related ratio of 18:0/16:0 and body mass in females (r = −0.57, *p* < 0.05; r = −0.51, *p* < 0.05, respectively).

## 4. Discussion

In this study, the FA composition of *m. biceps femoris* of autumn collected free-ranging edible dormouse in Croatia was determined for the first time. Thus, our results were compared with the results available in the literature for other animals. Specific omega-3 and omega-6 Fas were determined in *m. vastus lateralis* muscle of brown bears [24]. Authors determined that 18:3n-3 was the highest in the muscle in winter period. In another study, [25] authors found higher percentage of 18:2n-6 in the same muscle as well as in *m. gluteus superficialis* and *m. semitendinosus* in brown bears in spring compared to the autumn period. The *m. longissimus dorsi* in winter hibernating and winter active Yakut ground squirrels showed a decrease in the level of 16:0 a in relation to summer and autumn active ground squirrels [26].

Mice with low metabolic rates tend to live shorter lives compared to animals with high mass-specific metabolic rates [27]. Possibly, the theory of rate-of-living and oxidative-stress could explain the differences between individuals within a same species, as suggested by [28]. In this study, DHA% was significantly lower and MUFA% significantly higher with increased body mass in males (r = −0.62, *p* < 0.05; r = 0.66, *p* < 0.05). Phospholipids from the tissues of small mammal and bird species have a high content of DHA compared to large species due to higher metabolic activity [29]. It is known that n-3 PUFA and DHA contents decrease markedly as body size increases, in contrast to contents of total UFA and n-6 PUFA, which are independent of body mass [29]. When food is scarce on PUFAs, an increase of MUFA de novo synthesis (particularly in oleic acid, 18:1*cis*-9) has been observed in [30]. The relationship between the peroxidation index of skeletal muscle phospholipids and the maximum lifespan of mammal and bird species shows that both mammals and birds muscle membranes are more prone to peroxidation in the short living species [29], as described in the “membrane pacemaker” theory of aging [31]. A low peroxidation index value corresponds with a low DHA. When the peroxidation index is lower, the lifespan is longer. Edible dormice have a lifespan of 13 years, which is exceptionally long for a rodent [32].

In this study, we found an inversely proportional relationship between ARA, (females, r = −0.93, *p* < 0.05; males, r = −0.94, *p* < 0.05), DHA (females, r = −0.95, *p* < 0.05, males, r = −0.95, *p* < 0.05), and muscle lipid contents, whereas 18:1*cis*-9 and lipid contents showed a proportional relationship (females, r = 0.88, *p* < 0.05; males, r = 0.84, *p* < 0.05). Membrane phospholipids of type I fibers are characterized by a smaller proportion of palmitic acid (16:0) and a greater proportion of n-3 PUFA compared with type Iib fibers [33]. In this study, we found an inversely related ratio of 20:4n-6/20:3n-6 and body mass in males as well as an inversely related ratio of 18:0/16:0 and body mass in females (r = −0.57, *p* < 0.05; r = −0.51, *p* < 0.05, respectively). There is an inverse relationship of several Fas and ratios between 20:4n-6/20:3n-6 and 18:0/16:0 in skeletal muscle phospholipids to the percentage of body fat [34], which concurs with our results. In skeletal muscle, 20:4n-6 promotes myocyte growth and contributes to maintaining membrane fluidity and cell signaling. In this study, we found an inversely proportional relationship between 20:4n-6 and muscle lipid content. Some influence on intramuscular fat, especially in muscles displaying lower 20:4 content (*m. gluteus medius* vs. *m. semimembranosus*), could be exerted by the tag single nucleotide polymorphism (SNP) [35]. Literature states that the incorporation of 20:4n-6 into membrane phospholipids and influence in cell signaling could be muscle-specific [36]. Bears hibernate with only moderate hypothermia but with a drop in metabolic rate down to ~25% of basal metabolism. Results by [37] suggest that during hibernation, in bear muscle, there is a potential control of carbohydrate metabolism and protein sparing by actions of n-3 PUFA like DHA.

The structure of *m. biceps femoris* cells in regard with length and saturation of the membrane FA [38] are crucial for muscle physiology, especially during hibernation. Some research on small mammal’s muscle fiber-type distribution [39] showed an absence of type I fibers and the presence of mostly type II fibers. As for a hibernating animal, normothermic body temperature is of a high importance to maintain through both non-shivering and shivering thermogenesis [40,41]. Such muscle activity could result in oxidative stress [42] leading to lipid peroxidation, which most often affects PUFA [43] and likely influences FA composition in the muscle. The FA composition of *m. biceps femoris* in this study, especially regarding PUFA representation, which tended to be higher in males than females (Table 2), would be of interest for future research into dormice. The accumulation of 18:0 and 16:0 in *m. biceps femoris* in this study could be explained by specific fibers and muscle metabolism [44].

The FA 20:3 ∆5,∆11,∆14 that we identified in *m. biceps femoris* of edible dormice is found in various gymnosperm (conifer) species [45,46] and can be synthesized in gymnosperms and animals by elongation followed by “front-end” ∆5-desaturation (i.e., 18:2 ∆9,12 → 20:2 ∆11,14 → 20:3 ∆5,11,14) [47]. In addition to seeds, this FA also occurs in the leaf and wood lipids of Coniferophytes [48]. Sometimes, spruce bark can be peeled off at a specific height because of dormice feeding activity, which often leads to the death of the tree [49]. After reaching the appropriate body mass, the digestive system is cleaned and prepared for the winter by feeding exclusively on grasses and conifer needles [40].

Beech is a crucial factor determining the probability of reproduction as well as gaining sufficient body fat reserves prior to hibernation [50]. The total lipids of the beech seeds account for 40.7%, and the major components are triacylglycerols with 94.8%. Fatty acids such as 18:1*cis*-9 and 18:2n-6 are found in the highest representation (37.5% and 42.3%, respectively) [51]. Since, 18:2n-6 is an essential FA, it cannot be synthesized de novo by most animal cells and must be provided in the diet [52]. We found that males tended to contain a higher proportion of 18:2n-6 in *m. biceps femoris* than females (Table 1), which could be explained by the fact that males enter hibernation later than females [53]. Selecting beech nuts as the main source of food in autumn is especially true for hibernators and for adequate hibernation [54,55]; PUFA will thus be incorporated into depot fats and membrane phospholipids [50]. In deer mice (*Peromyscus maniculatus)* [56] and golden-mantled ground squirrels (*Spermophilus lateralis)* [57], diets supplemented with unsaturated Fas (especially 18:2n-6) enhanced hibernation. More animals were entering torpor, had a lower torpor body temperature, and longer bout duration during hibernation, thus corroborating our results.

In the present study, the ratio of 18:1/18:0 in females was 8.20, in comparison to 4.45 in males, which may indicate lower levels of desaturase activity in females [58]. Although, delta-9-desaturase indices were high in *m. biceps femoris* in edible dormouse in this study (Figure 2). The synthesis of mostly the *cis*-9 MUFA from their respective SFA by the introduction of the *cis*-9 double bond on the SFA is catalyzed by the delta-9-desaturase, which is encoded by the stearoyl coenzyme A desaturase (SCD) gene [23]. Results for SCD_i_17 and SCD_i_18 in females in this study are similar to the study of females Muskoxen muscle [22]. As discussed by [23], the large concentration of 18:1*cis*-9 and the relatively high SCD indices in dormouse muscle tissues might point to a low mobilization of the SCD products, specifically the 18:1*cis*-9. Authors further suggest [23] a selective salvage of the 18:1*cis*-9 during fat mobilization, which in this study is more evident in females dormice than males (Table 1). Since 18:1*cis*-9 is salvaged, this suggests that it can be selectively stored in dormouse muscle. This high activity of SCD_i_18 could be due to 13-fold greater stearoyl-CoA desaturase-1 expression in glycolytic compared to oxidative muscle [44]. The pattern of FA uptake, expression, and diet-induced changes in FA desaturating and elongating enzymes maintained higher FA unsaturation in *m. biceps femoris* in this study [44].

In this study, there were no significant differences in body mass between females and males, which was in accordance with findings reported by [59]. Both studies confirm the research of [60], who demonstrated sexual size monomorphism. Authors believe that the lack of sexual size dimorphism can be beneficial in the inter- and intra-sexual interaction, which may relate to passive mate-guarding. The observation that solitary species tend to show less sexual size dimorphism agrees with monomorphism [61]. The body mass of the edible dormice in this study was 113.4 ± 22.9 g and 126.2 ± 30.2 g (females and males), which is quite lower than the usual weight, around 200 g [5]. There is a positive correlation between pre-hibernation body mass and survival during winter [62]. Furthermore, fat content is reflected by pre-hibernation body mass [63]. This is especially true for juveniles, since they tend to have lower fat reserves prior to hibernation [64]. This study included younger animals that could stay awake longer to accumulate more fat for winter dormancy [16] and possibly delay hibernation onset.

Edible dormouse meat is traditionally eaten in some parts of Croatia as well as other parts of Europe. In this study, the muscle n-6/n-3 PUFA ratio, which was higher in males, does not fall within the FAO [65] recommendations. Concerning the n-6/n-3 PUFA ratio, a value between 5:1 and 10:1 has been reported in the International Society for the Study of Fatty Acids and Lipids (ISSFAL, [66]) and suggested by the joint Food and Agriculture Organization (FAO)/WHO committee. In this study, we found ratios of 4.69 ± 0.92 and 4.80 ± 0.80, in females and males, respectively. Furthermore, we also found that the PUFA/SFA ratio was lower when compared to recommended values from FAO [65] and [67]. The ratio of important FA groups SFA:MUFA:PUFA in this study was 2:0.5:0.5. The American Heart Association (AHA) dietary guidelines recognized the significance of the FA balance at approximately 1:1:1 for SFA:MUFA:PUFA [68]. Despite its importance from a global perspective, this may nevertheless be considered as less significant, as the meat of dormice tends to be consumed sporadically in Croatia and other countries.

## 5. Conclusions

Although we studied only one muscle, *m. biceps femoris*, the determination of specific Fas, such as DHA, ARA, and 18:1*cis*-9, could be important for maintaining membrane fluidity and cell signaling, especially during hibernation. The relatively high SCD indexes and the large concentration of 18:1*cis*-9 in dormouse muscle tissues might point to a selective salvage of the 18:1*cis*-9 during fat mobilization, which is more evident in female dormice. When food is scarce on PUFAs, an increase of MUFA de novo synthesis (particularly in oleic acid, 18:1*cis*-9) occurs, which also suggests feeding on beechnuts. 

Furthermore, the finding of FA 20:3 ∆5,∆11,∆14 suggests feeding on leaf and wood lipids of Coniferophytes. Moreover, selecting beech nuts as the main source of food in autumn is of importance regarding adequate preparation for hibernation. The small differences in FA composition of *m. biceps femoris* in males could be explained by the fact that males enter hibernation later than females. We confirmed sexual size monomorphism in edible dormice in Croatia.

## Figures and Tables

**Table 1:** Total fatty acid (FA) profile (% of total FA) in muscle (*m. biceps femoris*) of male and female edible dormouse (*Glis glis*).

	Females (*n* = 16)	Males (*n* = 14)	*p*-Value
Saturated FA			
14:0	0.78 ± 0.18	0.89 ± 0.15	0.402
15:0	0.018 ± 0.004	0.019 ± 0.004	0.567
16:0	13.2 ± 0.85	17.7 ± 0.74	0.782
17:0	0.05 ± 0.01	0.04 ± 0.01	0.743
18:0	5.90 ± 1.26	8.84 ± 1.10	0.746
20:0	0.07 ± 0.01	0.06 ± 0.01	0.254
Monounsaturated FA			
14:1*cis*-9	0.06 ± 0.01	0.06 ± 0.01	0.567
16:1*cis*-7	0.36 ± 0.08	0.20 ± 0.07	0.600
16:1*cis*-9	2.73 ± 0.61	2.51 ± 0.53	0.840
17:1*cis*-9	0.03 ± 0.01	0.04 ± 0.01	0.510
18:1*cis*-9	63.0 ± 6.50	47.7 ± 5.63	0.164
18:1*cis*-11	2.04 ± 0.20	2.32 ± 0.18	0.783
Polyunsaturated FA			
18:2 n-6	8.56 ± 3.57	11.4 ± 3.09	0.063
18:3 n-3	1.48 ± 0.22	0.93 ± 0.19	0.480
20:2 n-6	0.12 ± 0.05	0.14 ± 0.04	0.244
20:3 ∆5,∆11,∆14	0.10 ± 0.10	0.14 ± 0.08	0.835
20:3 n-6	0.11 ± 0.12	0.36 ± 0.10	0.998
20:3 n-3	0.03 ± 0.01	0.03 ± 0.01	0.196
20:4 n-6 (ARA)	0.89 ± 1.67	4.03 ± 1.45	0.380
20:5 n-3 (EPA)	0.02 ± 0.03	0.09 ± 0.02	0.226
22:4 n-6	0.04 ± 0.03	0.11 ± 0.03	0.890
22:5 n-6	0.03 ± 0.03	0.13 ± 0.02	0.190
22:5 n-3 (DPA)	0.11 ± 0.15	0.41 ± 0.13	0.423
22:6 n-3 (DHA)	0.38 ± 0.80	1.97 ± 0.69	0.406

ARA—arachidonic fatty acid; EPA—eicosapentaenoic fatty acid; DPA—docosapentaenoic fatty acid; DHA—docozahexaenoic fatty acid; statistical significance at levet *p* < 0.05.

**Table 2:** Fatty acids (FA) sums (% of total FA) and FA ratios of edible dormouse (*Glis glis*) muscle (*m. biceps femoris*).

	Females (*n* = 16)	Males (*n* = 14)	*p*-Value
Sums			
SFA	20.0 ± 1.27	27.6 ± 1.10	0.835
UFA	79.6 ± 1.27	72.5 ± 1.10	0.835
MUFA	68.2 ± 6.83	52.8 ± 5.92	0.189
PUFA	11.8 ± 6.07	19.6 ± 5.25	0.099
n-6 PUFA	9.76 ± 5.25	16.2 ± 4.54	0.119
n-3 PUFA	2.01 ± 0.90	3.43 ± 0.78	0.058
VLn-3PUFA	0.97 ± 0.24	1.73 ± 0.21	0.102
DHA + EPA	0.76 ± 0.16	1.42 ± 0.14	0.133
Ratios			
UFA/SFA	4.00 ± 0.20	2.65 ± 0.17	0.835
PUFA/SFA	0.66 ± 0.09	0.91 ± 0.09	0.059
18:1/18:2	8.2 ± 1.22	4.45 ± 1.30	0.068
AA/EPA	131 ±66.37	59.6 ± 57.48	0.624
AA/DHA	2.38 ± 0.29	2.06 ± 0.25	0.065
EPA/DHA	0.05 ± 0.03	0.07 ± 0.03	0.522
n-6/n-3	4.69 ± 0.92	4.80 ± 0.80	0.888

SFA—saturated fatty acids; UFA—unsaturated fatty acids; MUFA—monounsaturated fatty acids; PUFA—polayunsaturated fatty acids; VLn-s PUFA—very long PUFA from n-3 family; ARA—arachidonic fatty acid; EPA—eicosapentaenoic fatty acid; DPA—docosapentaenoic fatty acid; DHA—docosahexaenoic fatty acid; and statistical significance at levet *p* < 0.05.

## Data Availability

Data is contained within the article.
